# Blood–Brain Barrier Breakdown in Alzheimer’s Disease: Mechanisms and Targeted Strategies

**DOI:** 10.3390/ijms242216288

**Published:** 2023-11-14

**Authors:** Amer E. Alkhalifa, Nour F. Al-Ghraiybah, Julia Odum, John G. Shunnarah, Nataleigh Austin, Amal Kaddoumi

**Affiliations:** Department of Drug Discovery and Development, Harrison College of Pharmacy, Auburn University, 720 S. Donahue Dr., Auburn, AL 36849, USA; aea0068@auburn.edu (A.E.A.); nfa0007@auburn.edu (N.F.A.-G.); jgo0013@auburn.edu (J.O.); jgs0036@auburn.edu (J.G.S.); nna0005@auburn.edu (N.A.)

**Keywords:** Alzheimer’s disease, blood–brain barrier, BBB, drug development, transporters, receptors, mechanism

## Abstract

The blood–brain barrier (BBB) is a unique and selective feature of the central nervous system’s vasculature. BBB dysfunction has been observed as an early sign of Alzheimer’s Disease (AD) before the onset of dementia or neurodegeneration. The intricate relationship between the BBB and the pathogenesis of AD, especially in the context of neurovascular coupling and the overlap of pathophysiology in neurodegenerative and cerebrovascular diseases, underscores the urgency to understand the BBB’s role more deeply. Preserving or restoring the BBB function emerges as a potentially promising strategy for mitigating the progression and severity of AD. Molecular and genetic changes, such as the isoform ε4 of apolipoprotein E (ApoEε4), a significant genetic risk factor and a promoter of the BBB dysfunction, have been shown to mediate the BBB disruption. Additionally, receptors and transporters like the low-density lipoprotein receptor-related protein 1 (LRP1), P-glycoprotein (P-gp), and the receptor for advanced glycation end products (RAGEs) have been implicated in AD’s pathogenesis. In this comprehensive review, we endeavor to shed light on the intricate pathogenic and therapeutic connections between AD and the BBB. We also delve into the latest developments and pioneering strategies targeting the BBB for therapeutic interventions, addressing its potential as a barrier and a carrier. By providing an integrative perspective, we anticipate paving the way for future research and treatments focused on exploiting the BBB’s role in AD pathogenesis and therapy.

## 1. Introduction

Alzheimer’s disease (AD) is a chronic neurodegenerative disorder that predominantly affects the elderly, leading to cognitive decline, memory loss, and an impaired daily functioning [1]. As the most common cause of dementia, AD accounts for 60–80% of all cases, making it a significant health concern and the sixth leading cause of death for Americans aged 65 and above [2], with an estimated 6.7 million Americans are currently living with the disease [3]. Furthermore, with the aging of the global population, the prevalence of AD is on the rise, positioning AD as a formidable global healthcare challenge. In light of this growing health concern, the U.S. Food and Drug Administration (FDA) has granted an accelerated approval to a novel treatment of AD, Aducanumab, and the full approval to Lecanemab. These drugs are monoclonal antibodies targeting amyloid beta (Aβ) that is implicated in the AD pathology [4,5]. However, the other used medications, including the acetylcholinesterase inhibitors (galantamine, rivastigmine, and donepezil) and the N-methyl-D-aspartate (NMDA) antagonist, i.e., memantine, only provide symptomatic relief by improving memory and the ability to perform daily functions without curing the disease [6]. 

AD is primarily categorized into two forms: familial and sporadic AD. Familial or early-onset AD (EOAD), associated with autosomal dominant mutations, affects individuals under age 65 and represents 1% to 2% of all cases [7,8]. This form is typically characterized by mutations in specific genes, such as presenilin 1 (PSEN1), which are identified in up to 70% of familial AD cases, along with presenilin 2 (PSEN2) and amyloid precursor protein (APP) [8]. Conversely, sporadic or late-onset AD (LOAD) affects individuals over 65 years of age [9]. While age is considered the primary risk factor of LOAD, AD is a multifactorial disease without a single identified etiology. Several modifiable or non-modifiable risk factors, such as sex and family history, could increase the disease risk. Additionally, vascular risk factors such as hypertension are linked with the development and progression of the disease [10,11]. 

AD comprises two primary neuropathological hallmarks: extracellular amyloid-β (Aβ) accumulation and aggregated neurofibrillary tangles (NFTs) that are spread across the brain [12]. These pathological events induce neuronal atrophy and synaptic loss, culminating in neurodegeneration. Multiple hypotheses have been formulated to explain the development of AD, including the amyloidogenic cascade, tauopathy, neurovascular dysfunction, oxidative stress, and neuroinflammation [13]. Out of these, neurovascular dysfunction has received significant importance; several studies have implied that neurovascular dysfunction plays a vital role in the initiation and progression of AD, which suggests an association between alterations in cerebrovascular function and neurodegeneration [14]. In line with this, the two-hit vascular hypothesis of AD suggests cerebrovascular damage (hit 1) as an initial insult that is self-sufficient to initiate neuronal injury and neurodegeneration. Additionally, it promotes the buildup of Alzheimer’s Aβ toxin in the brain (hit 2) [15].

It is crucial to highlight the role of genetic factors in neurovascular dysfunction and their consequential influence on the development and progression of AD and cerebral amyloid angiopathy (CAA). CAA is a progressive Aβ build-up in the walls of small leptomeningeal and cortical arteries and cortical capillaries [16]. Amyloid plaques from CAA tend to accumulate in cortical and leptomeningeal vessels, whereas those caused by AD gather in the parenchyma [17]. The ε4 allele of the apolipoprotein E (ApoE) gene (ApoEε4) plays a crucial role in Aβ deposition as a senile plaque. It is associated with brain vascular damage, leading to AD and CAA pathogeneses [18,19]. Moreover, AD patients with homozygous ApoEε4 exhibit thinner capillary basement membranes and an increased plasma protein leakage into the cortex [20]. The ApoEε4 isoform has been linked to the blood–brain barrier (BBB) breakdown, reduced cerebral blood flow (CBF), neuronal loss, and behavioral deficits independent of Aβ [21].

The BBB dysfunction is increasingly recognized as a significant factor contributing to AD [22]. It has been suggested that the BBB breakdown may precede cognitive decline and neurodegeneration, highlighting the critical need for further exploration of the BBB’s role in AD and its potential as a therapeutic target [22]. The BBB endothelium is a specialized system of brain microvascular endothelial cells that separate circulating blood from the brain’s extracellular fluid, maintaining the brain’s homeostatic environment [23]. The BBB endothelium plays a crucial role in the protection and functioning of the brain, allowing the selective passage of nutrients and molecules essential for brain function while simultaneously preventing the entry of potentially neurotoxic substances [22].

In the present review, we delve into the dysfunction of the BBB within the neurovascular unit (NVU) framework during AD. We discuss the mechanisms underlying BBB leakiness that could contribute to the pathogenesis of the disease and the potential of targeting BBB as a therapeutic approach in AD.

## 2. Overview of the NVU and the BBB

**Figure 1:**
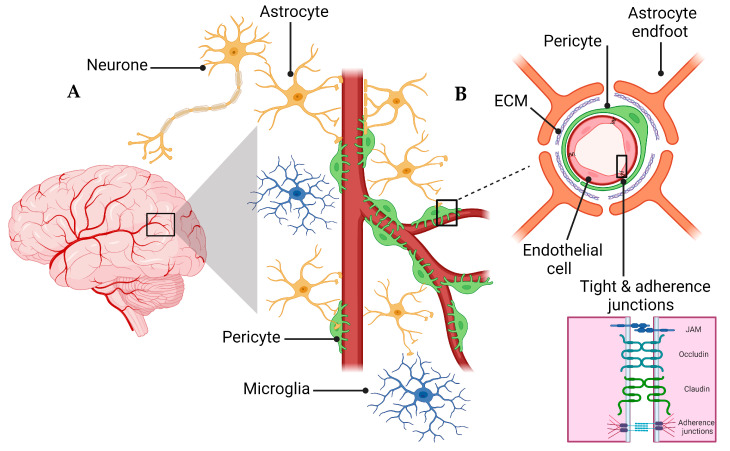
Schematic representation of the NVU (**A**) and the BBB (**B**). B is a magnification of the Box surrounding the capillary in A to demonstrate the cellular components of the BBB. The Box in B is magnified to show the connection between endothelial cells represented by tight and adherence junctions.

A schematic presentation of the NVU and the BBB are shown in Figure 1. The NVU is a crucial anatomical and functional unit that safeguards the homeostasis and optimal functioning of the central nervous system (CNS) [24]. Comprising several cell types, the NVU forms an interactive and dynamic system with its cellular constituents (Figure 1A). Neurons, the principal functional entities orchestrating signal transmission throughout the nervous system, are essential for the NVU [25]. Their close interaction with astrocytes, star-shaped glial cells, is pivotal for maintaining extracellular ion balance, facilitating synaptic transmission, and regulating CBF [26]. Microglia, the CNS’s resident immune cells, continually patrol the neural milieu and are ready to respond to alterations or threats [27]. Microglia participate in inflammatory responses and contribute to the NVU’s overall functionality by interacting with other cellular entities, including neurons and astrocytes [28].

The extracellular matrix (ECM), a non-cellular component of the NVU, provides structural support and influences cellular functions, including cell adhesion, migration, differentiation, and proliferation [29]. The NVU further comprises pericytes and endothelial cells, which are critical cellular components and contributors to the BBB’s integrity and function [30]. Pericytes, encapsulating the endothelial cells within capillaries, play a key role in vascular stability, angiogenesis, and the BBB’s permeability [31]. Understanding the interplay of these components within the NVU and their contribution to the BBB’s integrity is vital for investigating pathologies like AD that involve the BBB dysfunction [31].

The BBB is a highly selective semipermeable barrier crucial in maintaining brain homeostasis [22,32]. BBB acts as an essential protective and regulatory shield for the CNS, restricting the entry of neurotoxic substances from the blood circulation [33]. The BBB is more than a passive barrier; it performs a dynamic orchestration of the CBF to meet the metabolic demands of the neurons [34]. Its role is not only limited to maintaining CNS homeostasis by regulating crucial nutrients such as glucose and oxygen but also includes the selective removal of metabolic waste products from the brain [35]. In addition to these functions, the BBB is intimately linked to the endocrine system, responding to its signals and releasing substances that further influence brain function [35,36]. This dual role as an endocrine target and an endocrine secretory tissue underscores the BBB’s vital contribution in maintaining the delicate balance necessary for the proper functioning of the CNS [22,37]. 

The endothelial cells of the BBB are interconnected to form a polarized monolayer with unique luminal (apical) and abluminal (basolateral) compartments separating the brain parenchyma from the peripheral system (Figure 1B) [33]. These distinct compartments play a critical role in maintaining the physical and functional integrity of the BBB [33,38]. The endothelial cells contribute significantly to the BBB functionality [33]. Primarily, on the apical side, the membrane connecting CNS endothelial cells creates a paracellular barrier, restricting the diffusion of small hydrophilic molecules and ions [33,39]. Secondly, the passive and active receptors, channels, and transport proteins located on the luminal and abluminal sides govern the transportation of endogenous molecules into and out of the brain. Lastly, the endothelial cells function as a communication medium between the CNS and the peripheral system by controlling the migration of circulating immune cells into the brain’s microenvironment [33,39]. The BBB’s endothelial junctions are integral in ensuring tissue integrity and regulating vascular permeability. Endothelial cells of the BBB form a tightly sealed monolayer that is interconnected through a junctional complex comprising tight junctions (TJs) and adherens junctions (AJs) (Figure 1B) [34]. TJs form the outermost boundary between endothelial cells in the BBB, functioning as a boundary between apical and basolateral plasma membrane domains and sealing the paracellular space to control protein diffusion and cell trafficking [38]. TJs and AJs comprise transmembrane molecules such as occludin, claudins (claudin-3, -5, and -12), zonula occludins (ZO-1, ZO-2, and ZO-3), and junctional adhesion molecules (JAMs) [40]. AJs are commonly intermingled with TJs within the structure of the BBB. The endothelial-specific integral membrane protein, VE-cadherin, is connected to the cytoskeleton through a set of proteins known as catenins, which are part of the armadillo protein family [41]. Within the BBB, the expression and proper localization of β-catenin, χ-catenin, and p120cas are indispensable for the functional integrity of the AJs and, by extension, the overall functionality of the BBB [42,43].

## 3. BBB Dysregulation with Aging

The dysregulation of BBB in aging involves both a loss of selective transport mechanisms and a reduction in structural integrity [44]. This is exemplified by a global shift in the pattern of protein transcytosis, transitioning away from a receptor-mediated transport (RMT) to an increased caveolar transcytosis, which allows the entry of potentially neurotoxic proteins such as albumin and fibrinogen that are otherwise restricted in youth [44,45]. This change in the BBB’s permeability is implicated in the pathophysiology of neurodegenerative diseases, including AD, by facilitating neuroinflammation through unregulated protein entry [46,47]. Moreover, the age-related decrease in pericyte coverage further compromises the BBB, impairing blood flow and neuronal function [48]. The observed upregulation of endothelial alkaline phosphatase, namely ALPL, in AD patients, suggests it to be a potential therapeutic target, as its inhibition could modify the BBB’s permeability and influence the disease progression [49]. Overall, these changes underscore a “two-hit” hypothesis for vascular contributions to AD, where both pericyte loss and dysfunctional transcytosis act synergistically with other pathogenic factors like Aβ accumulation. Understanding the evolving nature of the BBB’s communication with the periphery is crucial for deciphering the impact of aging on the brain in health and disease [49].

## 4. BBB Dysfunction in AD 

The BBB breakdown in AD has been confirmed by more than 20 independent post-mortem human studies showing brain capillary leakages and the perivascular accumulation of blood-derived fibrinogen, thrombin, albumin, immunoglobulin G (IgG), and hemosiderin deposits, as well as the pericyte and endothelial degeneration, loss of BBB TJs, and red blood cells (RBC) extravasation. Although the BBB breakdown has been associated with AD, it can occur independent of the disease with aging [50]. As the BBB disruption has been associated with aging, a cognitive impairment is absent in mice unless accompanied by inflammation [51]. The BBB breakdown is linked to inflammation and dementia in aging humans with a mean age of 70 years [52], vascular cognitive impairment and dementia (VCID), and AD [53]. The disruption could be mediated by carrying genes such as ApoEε4, inflammation, stress, and the accumulation of time-dependent compounds such as reactive oxygen species (ROS) or Aβ in AD [54,55]. 

Systemic inflammation disrupts the BBB as well. This disruption depends on sex, other comorbidities, genetics, and gut dysbiosis [56]. Systemic inflammation and infections affect cellular signaling between immune cells and endothelial cells. This effect might be mediated by soluble inflammatory markers, as seen when treating mice with lipopolysaccharide (LPS) [57]. LPS alone did not alter the transendothelial electric resistance (TEER), yet serum from LPS-treated mice reduced TEER and sphingosine-1-phosphate (S1P) and affected the sphingolipid metabolism, which is a mechanism of the BBB breakdown [57].

### 4.1. Effect of Comorbidities on BBB 

The BBB disruption is related to multiple comorbidities, including vascular comorbidities such as atherosclerosis, where both diseases are characterized by inflammation and vascular dysfunction [58]. Small vessel dysfunction is responsible for the BBB breakdown, CBF and Aβ clearance reduction, and neuronal dysfunction [58,59]. However, findings from the Baltimore Longitudinal Study of Aging (BLSA) Cohort, which investigated the relationship between Alzheimer’s and atherosclerosis, found no association between the degree of Alzheimer’s pathology and atherosclerosis in the aorta, heart, and intracranial vessels, though only intracranial atherosclerosis correlated with dementia [60]. 

Moreover, comorbidities, including Type 2 Diabetes Mellitus (T2DM), hypertension, obesity, sleep disorders, and hypercholesterolemia, are increasingly recognized as contributing factors in the disruption of the BBB and the progression of AD [61,62]. In the case of T2DM, which is characterized by insulin resistance and chronic hyperglycemia, it causes endothelial dysfunction and inflammation that could ultimately compromise the BBB’s integrity. These alterations potentially facilitate the entry of neurotoxic molecules like Aβ, thereby contributing to AD pathology [63,64]. Furthermore, hypertension manifests as an elevation in systemic blood pressure, triggering vascular remodeling and neuroinflammation. These mechanistic pathways weaken the BBB, thereby augmenting microvascular damage in AD [65].

Obesity, especially as a part of the metabolic syndrome, often precipitates systemic inflammation and endothelial dysfunction, contributing to a compromised BBB function [66]. Sleep disorders, particularly those impacting the glymphatic system, are emerging as critical players in hindering the efficient clearance of neurotoxic products such as Aβ, ultimately fostering an environment conducive to AD progression [67]. Similarly, hypercholesterolemia, marked by elevated cholesterol levels, is associated with increased Aβ production and impaired clearance mechanisms, eventually forming amyloid plaques [68].

The cumulative impact of these comorbidities cannot be overlooked, as they often co-occur and may have synergistic effects on BBB dysfunction and AD pathology. These comorbidities exacerbate the degradation of TJs, thereby compounding the risk of BBB dysfunction and the subsequent development and progression of AD [69,70,71,72]. The interplay between the degradation of TJs by proteases and the risk factors for AD suggests a vicious cycle where the compromised barrier function of the BBB can lead to further neuronal damage and progression of AD. At the same time, the disease state promotes a further BBB breakdown [72]. Understanding these mechanisms is crucial for developing targeted therapies that can preserve TJ integrity and protect against the progression of AD [73]. 

Indeed, understanding the molecular and cellular interplay between these comorbid conditions and the BBB’s integrity can offer novel insights into therapeutic avenues. Addressing these comorbidities may serve as a preventative strategy and enhance the efficacy of existing BBB-targeted therapies, thereby improving clinical outcomes in AD patients.

### 4.2. ApoE and BBB Dysfunction in AD

ApoE is mainly expressed in astrocytes and microglia in the brain; its functions are associated with the endocytosis of lipoproteins, the deposition and transport of Aβ, membrane integrity, neurotoxicity, and synaptic plasticity [19]. It is produced in the brain, liver, kidneys, skin, adipose tissue, and many other organs [74]. Within the CNS, ApoE is produced de novo separately and independently from peripheral ApoE [75]. 

ApoE protein has three different isoforms: ApoEε2, ApoEε3, and ApoEε4. These isoforms have functional and structural differences, inconsistencies, and discrepancies in their interaction with low-density lipoprotein (LDL) receptors [74]. Within the cell, ApoE plays a role in cellular processes involving the maintenance of the cytoskeleton, mitochondria, and dendrites, leading it to play a prominent role in the overall health of neurons [74]. ApoE amino acids vary at locations 112 and 158 on chromosome 19, and there are six different genotypes, i.e., three homozygous and three heterozygous [76]. ApoEε2 is protective against the pathology of AD [77]. ApoEε2 expression clears Aβ normally and may suppress inflammation, which can protect against AD [76]. Moreover, ApoEε2 increases the clearing and metabolism of lipids, which is related to better memory and cognitive longevity [78]. It is also associated with an increased neural plasticity. Alternatively, individuals with the ApoEε4 isoform display a less efficient regenesis of neural connections after injury due to a decreased ability to redistribute cholesterol and proteins [19]. 

To investigate the role of ApoEε2 in AD, using post-mortem human cortices, Fernández-Calle, and colleagues reported that the abundance of ApoEε2 and its ability to bind to LDLRs increased the efficiency of Aβ clearance. In addition, they found that the expression of mRNA molecules related to ECM increased, which could contribute to the protection of the BBB’s integrity. These effects of ApoEε2 point toward its protective abilities against AD [79]. ApoEε3, the most common form of the gene, on the other hand, does not present a risk for or protection from AD relative to the ε4 and ε2 alleles, respectively. ApoEε3 protein functions well in binding to LDL, very low-density lipoprotein (VLDL), and low-density lipoprotein-related protein-1 (LRP1) receptors, which play a role in clearing Aβ [79].

The most heavily researched isoform is ApoEε4. ApoEε4 is associated with an earlier onset of AD due to a higher plaque density earlier in life [19]. ApoEε4 likely contributes to AD pathology by increasing neurotoxicity and decreasing the protection of the CNS [75]. However, the mouse apoE gene exists only in one isoform and is located on chromosome 7 rather than 19. For this reason, mice are often artificially introduced to human ApoE to make the study applicable to human AD [79]. ApoEε4 correlates with a decreased BBB integrity and cognitive impairment due to disturbed homeostasis and neurotoxin extravasation into the brain [80]. ApoEε4 knock-in mice displayed the loss of pericytes and a subsequent BBB degradation as they aged [81]. This loss of pericytes was also found in human ApoEε4 carriers [75]. In another study with ApoEε4 knock-in mice, it was found that the cyclophilin A matrix metalloproteinase 9 (CypA-MMP9) degradation pathway was activated in the pericytes of affected individuals, which led to a decrease in TJ proteins and the breakdown of the BBB that correlated with the leakage of neurotoxic proteins [21]. In AD patients who carried the ApoEε4 allele, post-mortem tissue analysis and living CSF analysis confirmed the activation of the CypA-MMP9 degradation pathway [82]. ApoEε4 carriers demonstrate reduced CBF, BBB integrity, and cognitive function without a change in Aβ [83]. These results are significant because they suggest that ApoEε4 plays a role in AD independent of Aβ and functions in mechanisms that should be investigated further as potential therapies [21]. However, ApoEε4’s role in the deposition of Aβ and its contribution to the AD pathology has also been described. ApoEε4 inhibits the clearance and metabolism of Aβ, which increases the deposition of the plaques, leading to the earlier manifestation of cognitive symptoms of AD [78].

### 4.3. Tight Junctions’ Role in BBB Health and Disease

The barrier function of TJs in the BBB is multifaceted. They restrict the intercellular space, preventing the unregulated diffusion of substances between the blood and the brain. This role is critical in preserving the specialized environment required for an optimal neural function [84]. Concurrently, TJs maintain cellular polarity by ensuring an asymmetrical distribution of proteins across the cell, a decisive factor for a directional transport across the endothelium [84]. On the paracellular front, TJs exert tight control over the diffusion pathways by leveraging an array of integral proteins, such as claudins, occludin, and JAMs. The specific assembly of these proteins determines the permeability properties of the junctions, allowing selective passage of ions and molecules while safeguarding against harmful substances [85]. For the transcellular route, the asymmetrical distribution of transporters and channels underpins a regulated transport mechanism, ensuring that essential nutrients reach the brain while metabolic waste products are efficiently evacuated. The concerted action of these transcellular elements with TJs establishes the BBB as a dynamic regulatory interface, which is adept at adjusting to physiological needs and protecting against pathological insults. Collectively, these mechanisms underscore the sophisticated control that TJs exert on the BBB’s function. Understanding the intricate balance between the transcellular and paracellular pathways and the role of TJs in this equilibrium are pivotal in elucidating the pathophysiology of neurodegenerative diseases like AD. It can guide the development of therapeutic strategies aimed at bolstering the BBB’s integrity.

In AD, one of the critical pathways by which TJs can be compromised involves the activity of matrix metalloproteinases (MMPs) and other proteases [86]. MMPs are a family of zinc-dependent endopeptidases capable of degrading various components of the extracellular matrix (ECM). They are known to modulate the permeability of the BBB by targeting TJ proteins [87]. MMPs, particularly MMP2 and MMP9, have been demonstrated to cleave occludin, claudins, and ZO proteins, leading to the disassembly of TJs and an increased BBB permeability [87]. The activity of MMPs is tightly regulated under normal physiological conditions but can be upregulated in response to inflammation, ischemia, and oxidative stress, which are common in the AD pathology [86].

Moreover, a study has indicated that during epileptic seizures, glutamate release can escalate MMP2 and MMP9 levels at the BBB [88]. This elevation in MMP activity correlates with decreased TJs, leading to an increased barrier permeability. The study implies that cytosolic phospholipase A2 (cPLA2) plays a significant role in this process and may serve as a therapeutic target to ameliorate barrier dysfunction. Thus, in AD, where the BBB leakage can exacerbate disease progression, targeting cPLA2 to mitigate MMP-induced TJ degradation could be a viable strategy. This approach not only holds the potential for improving epilepsy treatment but also for addressing other neurological disorders where the BBB leakage is a contributing factor. The relationship between TJ degradation by MMPs and AD risk factors underscores the importance of maintaining the BBB’s integrity, as the breach of this barrier can lead to neuronal damage and further propagate the cycle of neurodegeneration [88]. Other proteases, such as the disintegrin and metalloproteinase (ADAM) family and serine proteases, also contribute to the remodeling of the BBB by cleaving components of the TJs [89]. The resultant enhanced BBB permeability can facilitate the entry of neurotoxins into the brain parenchyma, promoting neuroinflammation and neuronal injury [90].

### 4.4. Transporters’ Role in BBB Health and Disease 

The functionality of the BBB is partly enabled by the presence of diverse influx and efflux transporters expressed in the endothelial cells, notably from the solute carrier (SLC) and ATP-binding cassette (ABC) superfamilies [91,92,93]. The BBB restricts drug access to the brain by permitting only lipophilic molecules of low molecular weight into the brain via the transcellular pathway from the bloodstream, where most researchers consider a cut-off point of 400–600 kDa. However, deviations from this cut-off point have been published [94]. Small, lipid-soluble drugs, such as antidepressants, penetrate the BBB via passive diffusion across the BBB [38]. Transcytosis transports macromolecules, such as insulin and amino acids [38]. Efflux transporters counteract the passive diffusion by forcing foreign substances, toxic metabolites, and other waste products out of the brain [38]. 

In contrast, recombinant proteins, therapeutic antibodies, and nucleic acid drugs are too large to cross the BBB [38]. The BBB disruption could be due to, at least in part, the modulation of transporters’ function or expression, accumulating waste products, and the inadequate nutrient delivery to the brain [38]. Overall, more than 50 transporters are expressed in the BBB, and their modulation by aging or AD could alter the BBB function, thus contributing to AD pathology [95]. In this review, we report the most studied transporters in AD.

#### 4.4.1. SLC Transporters

SLC transporters are essential to regulate neurotransmitters and amino acid concentrations and drug influx into the brain [91]. For example, genetic inhibition of SLC39, a zinc transporter, reduced the Aβ pathology in the drosophila model of AD by reducing Aβ_42_ fibril deposits and neurodegeneration and improving the cognitive performance of the Aβ_42_-transgenic flies, which suggests that zinc homeostasis is necessary for the clearance of Aβ [96]. Astrocytic glutamate transporters, including the glutamate–aspartate transporter (GLAST) and glutamate transporter-1 (GLT-1), are responsible for the regulation of glutamate in the brain by taking up glutamate and limiting excitotoxicity [97]. GLT-1, which has been linked to AD [97], has the same effect as the overexpression of excitatory amino acid transporters (EAATs) in AD [98]. In addition, in AD, glucose transporters, particularly the endothelium-GLUT1, are downregulated early in the disease [99,100]. 

GLUT1-deficient APPSw mice have a lower CBF and an early BBB breakdown, as demonstrated by the increased fibrin and IgG extravasation and accelerated cerebral β-amyloidosis evident by the increased cortical and hippocampal Aβ load [101]. Several studies have shown that AD patients have a less glucose uptake into the brain, as determined using F-2-fluoro-2-deoxy-d-glucose- positron emission tomography (FDG-PET) imaging [38]. In addition, it has been reported that, in GLUT1-deficient APPSw mice, the loss of GLUT1 initiated the BBB breakdown by reducing TJs’ expression, increasing Aβ cerebral deposition, and causing cognitive impairment [102]. On the other hand, GLUT-1 conditional knockout from the astrocytes using tamoxifen-inducible Cre/LoxP mice model (GLUT1^ΔGFAP^ mice) reduced glucose uptake and glycolysis while preserving the total ATP production and improving the cognitive function. These results indicate that GLUT1 knockout in the astrocytes impairs the astrocytic glucose availability, enhances the brain glucose utilization, and reprograms the systemic glucose metabolism for efficient glucose handling, thereby collectively promoting cognitive function [103]. 

Furthermore, the dysregulation of amino acid transporters has been reported. For example, in isolated microvessels from brains of 5xFAD, an AD mouse model, the expression of the transporter alanine/serine/cysteine/threonine transporter (ASCT1) is two times higher than that in the isolated brain microvessels of the wild-type mouse [104]. On the other hand, the large neutral amino acids transporter small subunit 1 (LAT1) was less expressed in the 5xFAD mouse brain microvessels. However, when compared to humans, the expression of cerebrovascular LAT1 was not different between cognitively normal and AD subjects, although both species had high levels of phenylalanine in the brain, suggesting differences in LAT1 function in the microvessels between mice and humans [104]. Besides glucose and amino acid transporters, the expression of lactate transporters is altered in AD. Researchers have found that the expressions of lactate transporters, namely, the monocarboxylate transporters 1, 2, and 4 (MCT1, MCT2, and MCT4), are decreased in APP/PS1 mouse brains, causing a deficient lactate content in the neurons, and thus, reducing the memory formation and synaptic transmission in the hippocampus [105].

#### 4.4.2. ABC Transporters

ABC transporters are highly expressed by brain endothelial cells in the BBB. Because of their widespread tissue distribution, they are essential players in cellular homeostasis, but they may also be causative or contributing factors in AD [106]. ABC transporters use ATP to move their substrates across membranes of organelles, cells, and tissues. The substrates of ABC transporters involved in AD include cholesterol, sterols, lipids, peptides, metabolites, and xenobiotics such as toxins and a wide range of therapeutic drugs [107]. For example, ABCA1 is thought to play a role in AD pathogenesis because it enhances cholesterol and phospholipid efflux into ApoE particles and impacts the apolipoprotein lipidation and ApoE brain levels [107]. ABCB1 or P-glycoprotein (P-gp) is a member of the ABC superfamily responsible for the brain’s efflux of its substrates back into the circulation. It is highly expressed in the endothelial cells of the BBB, thus contributing to the clearance of toxins and metabolic waste, including Aβ [108]. In the AD pathology, P-gp expression is reduced [109], causing a lower support of the BBB function and the accumulation of Aβ in the brain. Reports have shown that, in P-gp-null mice, injected Aβ_40_ and Aβ_42_ into the brain were cleared to a lower extent than in the wild-type mice expressing normal P-gp levels [110]. 

Similarly, in humans where P-gp is reduced in the hippocampus, proven by the reduced BBB-mediated clearance of the P-gp probe, [11C]-verapamil, observed using PET imaging [109]. The same result was confirmed in frozen human tissue, isolated microvessels, and by analyzing the protein expression using targeted liquid chromatography-mass spectrometry in age-matched controls and AD subjects. Findings consistently demonstrated a P-gp downregulation with age and AD pathology in the hippocampus and cortex and only with age in the cerebellum [109]. Other ABC-transport proteins affected by the Aβ accumulation are ABCG2 and ABCC1; this alteration was seen in the cerebral vessels of TgF344-AD rats [111]. ABCG2 has been shown to prevent blood Aβ influx into the brain in ABCG2-null mice. The ABCG2-null mice had higher CY5.5 signals in their brains after the intravenous injection of Cy5.5-labeled Aβ_1-40_ or scrambled Aβ_40-1_ compared to those in wild-type mice [112]. Furthermore, the transporter ABCC1 plays a role in clearing Aβ from the brains of mice. ABCC1-knockout APP/PS1 mice had a higher Aβ load and a larger size of Aβ-plaques, and the activation of ABCC1 by thiethylperazine reduced the Aβ load in APP/PS1 mice compared to that in the vehicle-treated mice [113].

#### 4.4.3. MFSD2A Symporter

Major Facilitator Superfamily Domain Containing 2a (MFSD2a) is a sodium-dependent lysophosphatidylcholine (LPC) symporter that is expressed on the endothelial cells of the BBB [114]. It is responsible for lipid transport, particularly of docosahexaenoic acid (DHA). Although the expression of MFSD2a in the BBB was unchanged in AD patients, its serum expression was reduced progressively with the AD pathology [115]. Therefore, the use of MFSD2a as a blood biomarker for AD has been proposed [115]. Other studies on E4FAD mice and humans showed a lower MFD2a expression in brain homogenates of those carrying the ApoEε4 allele compared to that in ApoEε3 carriers. The ApoEε4 genotype was associated with reduced MFSD2a expression and DHA levels [116]. A lower expression of MFSD2a will decrease DHA levels in the brain, resulting in neuronal death, cognitive dysfunctions, and microcephaly [114,117]. It has been shown that MFSD2a knockout mice upregulated the transcytosis, causing the leakiness of the BBB [118]. Research on male Sprague Dawley rats with chronic cerebral hypoperfusion (CCH) showed that the overexpression of MFSD2a reduced the BBB damage and the cognitive decline. This effect was not due to an increased TJs’ expression but due to the inhibition of transcytosis associated with an increased MFSD2a [119].

#### 4.4.4. LRP1

LRP1 is a receptor responsible for the Aβ clearance across the BBB [120]. In the BBB, LRP1 is expressed in both pericytes and endothelial cells [120]. In the brains of mice of the APPSwe mouse model of AD and AD human patients, Aβ accumulated in pericytes associated with brain capillaries [120]. Using freshly isolated cortical mice brain slices, the Aβ uptake by pericytes was reduced when co-treated with the LRP1 antibody by 17% [120]. This was confirmed in the LRP1 conditional knockout (Lrp1^lox/lox^; Cspg4-Cre) mouse model, where the Aβ42 uptake in pericytes was also reduced [120]. Endothelial LRP1 has been shown to work with P-gp to efflux Aβ across the BBB, which is mediated by the protein phosphatidylinositol binding clathrin assembly (PICALM) [121]. PICALM directs the Aβ-LRP1 complex to endosomes that express P-gp, causing an Aβ efflux [121]. Other studies showed that the LRP1 ablation in a Lrp1^lox/lox^; Slco1c1-CreER^T2^ mouse decreased the BBB’s integrity by reducing the expressions of TJs and P-gp compared to those in Lrp1^lox/lox^ controls at 12 months of age [122]. In addition, the BBB dysfunction due to the reduced LRP1 expression could be mediated by the CypA–MMP9 pathway. Mice with LRP1 knockout (Lrp1^lox/lox^; Tie2-Cre) demonstrated an activated CypA–MMP9 pathway in the BBB endothelial cells, causing a reduction in TJs’ proteins and collagen IV basement membrane protein, hemosiderin deposition, and neurodegeneration [123]. Blocking CypA by Debio-025 (a non-immunosuppressive CypA inhibitor) for 30 days reduced the MMP9 activation, restored the endothelial TJs, and reduced the neurodegeneration in Lrp1^lox/lox^; Tie2-Cre mice compared to untreated mice [123].

Besides its role in the Aβ clearance across the BBB, LRP1 also has a role in generating Aβ [120]. LRP1 can interact with the APP and influence its processing. When APP is cleaved by β-secretase and γ-secretase, Aβ is generated. The interaction between LRP1 and APP can potentially enhance the amyloidogenic pathway, leading to an increased production of Aβ peptides [124]. The dualistic nature of LRP1 in the AD pathology suggests a delicate balance in its functions that could tip the scales toward either neuroprotection or neurodegeneration. Therapeutic strategies targeting LRP1 must, therefore, carefully consider these opposing roles [120,124]. 

In a pivotal study by Storck et al., the first known brain endothelial-specific Lrp1 knockout model revealed that the conditional knockout of Lrp1 in the cerebrovascular endothelium of C57BL/6 mice significantly inhibited the cerebral clearance of 125I-Aβ_42_, establishing LRP1 as a critical transporter in mediating the Aβ efflux from the brain. This effect was further evidenced in the 5xFAD mouse model, where endothelial-specific deletion of Lrp1 resulted in a heightened brain amyloid burden and pronounced cognitive impairments. These findings underscore the vital function of Lrp1 in the Aβ clearance at the BBB and suggest that disruption of this pathway can lead to the accumulation of Aβ and subsequent AD-related behavioral deficits [125].

Modulating LRP1 activity may require a balanced approach that promotes its role in clearance without inadvertently increasing the Aβ generation, potentially involving therapies that can specifically enhance the efflux function of LRP1 or stabilize its beneficial interactions without affecting the endocytic pathways that lead to the Aβ production. Moreover, another study unveils compelling in vivo evidence that disrupting LRP1-mediated endocytosis shifts the APP processing towards non-amyloidogenic pathways, thereby reducing the amyloidogenic processing and decreasing extracellular Aβ and plaque formation in an AD mouse model. Despite a decrease in the Aβ clearance, the inactivation of LRP1 paradoxically leads to a low Aβ accumulation due to an enhanced alpha-secretase activity and an inhibited Aβ generation post-endocytosis. This counterintuitive outcome demonstrated, through both in vitro and in vivo experiments, that a nuanced mechanism where a diminished LRP1 endocytosis is associated with a reduced Aβ deposition in AD, providing intriguing avenues for therapeutic exploration [126].

#### 4.4.5. RAGEs

The receptor for advanced glycation end products (RAGEs) has also been linked to AD pathology. RAGEs are responsible for the Aβ and neuropeptide transport across the BBB [127]. In AD, RAGE transport of Aβ through the BBB is disturbed, whereas the RAGE expression is increased, leading to an increase in the brain influx of Aβ [127]. Findings with isolated C57BL/6 mouse primary astrocytes, neurons, and endothelial cells in a BBB model showed that suppressing RAGEs with siRNA transfection led to an increase in the BBB model’s integrity as determined by an increased TEER when compared to controls treated with Aβ [127]. This result suggests that the interaction of Aβ and RAGE reduces the BBB’s permeability and that reduces the RAGE–Aβ interaction, thereby enhancing the BBB function [127]. In addition, the in vitro exposure to Aβ oligomers caused the upregulation of RAGEs in the mouse cerebrovascular endothelial cells, bEnd3, and in activated matrix metalloproteinase 2 (MMP2) and MMP9 [128]. Moreover, using the diabetes mellitus type 2 mouse model db/db (BKS. Cg m +/+ Lepr^db^/J), the blocking of RAGEs with FPS-ZM1 1.0 mg/kg reduced the Aβ efflux across the BBB, inhibited the nuclear factor-κB (NF-κB) signaling and neuronal apoptosis, increased the synaptic plasticity, and ameliorated the cognitive function [129]. 

#### 4.4.6. Multidrug Resistance-Associated Proteins (MRPs)

Multidrug resistance-associated proteins (MRPs) are a family of ABC transporters that regulate drug efflux across the BBB [130]. MRPs, such as MRP1, MRP2, and MRP4, are expressed on the luminal and abluminal membranes of brain capillary endothelial cells, playing a role in maintaining the homeostasis of the CNS by restricting the entry of potentially harmful substances and metabolites [131]. In AD, accumulating evidence suggests a potential link between the MRP dysfunction and the AD pathology. For instance, MRP1 and MRP2 expression levels have been observed to be downregulated in post-mortem AD brains [132]. This decreased expression could contribute to an impaired efflux of neurotoxic substances and Aβ peptides, potentially exacerbating AD pathology [113]. Moreover, recent studies indicate that certain MRPs may be involved in clearing Aβ peptides from the brain, with the dysregulated MRP function potentially contributing to Aβ accumulation and neurotoxicity [15], given the potential connection between the MRP dysfunction and AD pathology.

## 5. BBB Breakdown Mechanisms

**Figure 2:**
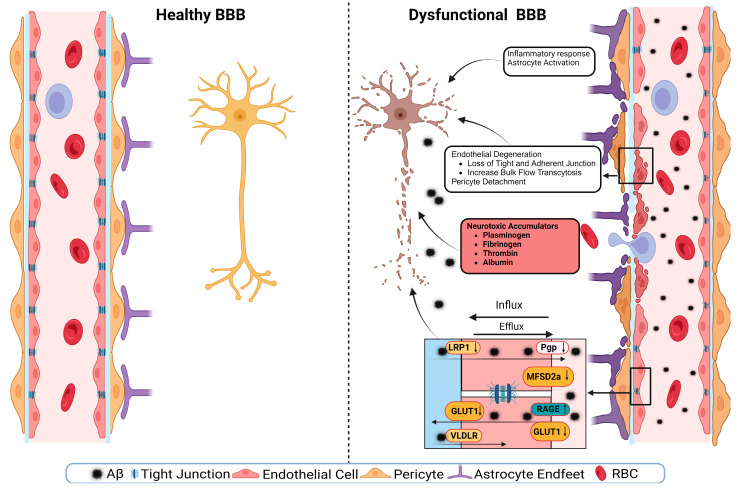
A representative scheme demonstrates a healthy BBB and a dysfunctional BBB.

A schematic presentation of the discussed mechanisms is presented in Figure 2. The disruption of the BBB could impair any of the NVU cellular components. The disruption of endothelial cells might result in a decreased TJs’ expression. It has been shown that occludin, claudin-5, and ZO1 expressions are lower in the Aβ-laden capillaries of patients with capillary CAA [38], implying that reduced TJs may increase the vascular permeability in an AD brain. Multiple cellular signaling pathways, such as calcium signaling, could mediate the disruption of TJs. In AD, the RAGE–Aβ_42_ interaction disrupts TJs via a calcium-calcineurin signaling pathway [133]. In addition, in the monolayer culture of bEnd3 cells, adding Aβ_42_ induces structural alterations in ZO1 [133]. Other TJ proteins, such as claudin-5 and occludin, were also structurally altered, and their expression was reduced by Aβ_42_ [133]. This result was confirmed as neutralizing antibodies against RAGEs and calcineurin and MMP inhibitors prevented Aβ_42_-induced changes in the ZO1 expression [133]. Furthermore, the expression of TJs is negatively correlated with Aβ_40_ levels in cortical areas and positively correlated with synaptic markers in patients with AD, highlighting the role of TJs in the AD pathology development [134]. 

Transport protein expression alterations in endothelial cells could be a mechanism or a consequence of BBB disruption [54]. In AD, the failure of Aβ clearance through transport across the BBB is caused by decreased levels of LRP1 and P-gp and an increased RAGE expression. For example, in an in vitro experiment, a high cholesterol level decreased the LRP1 expression, increased the RAGE expression, and increased Aβ40 levels in cerebral microvascular endothelial cells [135]. Other transport disruptions include a reduced expression of the GLUT1 transporter [54,136]. This effect was seen with a lower expression of GLUT1 in endothelial cells but not in astrocytes of the BBB in GLUT1-deficient APP_sw_-mice [102]. The LRP1 protein is vital to maintain the BBB’s integrity; it acts as a co-activator of peroxisome proliferator-activated receptor gamma (PPARγ), transports cholesterol associated with ApoE [137], plays a role in glucose metabolism [138], and interacts with Aβ to transport it across the BBB [137]. The LRP1 activation can stimulate PPARγ and increase TJs’ expression. This effect has been investigated in mouse models where a selective brain endothelial LRP1 knockout reduced the expression of TJ proteins and P-gp, increased the MMP activity, and decreased the TEER, leading to endothelial cell disruptions [122]. Endothelial cell disruption causes neuroinflammation due to neurotoxic substances infiltrating into the brain, causing inflammation and neurodegeneration. Fibrinogen leakiness in the brain activates the macroglia and initiates inflammation [139,140]. In AD, the same effect was seen in the 5xFAD mouse model, where fibrinogen caused synaptic loss by increasing ROS and microglial activation [140].

Aside from endothelial cell disruptions, neuroinflammation and the activation of glial cells can further drive AD pathology. Microglial activation can release pro-inflammatory factors, resulting in neuroinflammation [141]. Although inflammation is a protection mechanism, an overly aggressive inflammatory response can cause or contribute to tissue damage. In AD, astrocyte degeneration causes the BBB disruption. Using tamoxifen-astrocyte-depleted mice, large molecules such as fibrinogen and smaller molecules such as cadaverine, an exogenous labeled small molecule, were detected in the brain as soon as the astrocytes were depleted, which was accompanied by reduced levels of TJ proteins, and lower expression of GLUT1 [142]. Aquaporin-4 (AQP-4), a water channel facilitating bidirectional water transfer, is expressed by astrocytes at the BBB [56]. In AD, the expression of AQP-4 is reduced, which causes a reduction in the Aβ clearance through the glymphatic system [143]. AQP-4’s role in maintaining the BBB function has been shown in intracerebral hemorrhage mouse models where the wild-type-induced intracerebral hemorrhage mice had lesser BBB dysfunction and perihematomal edema compared to those observed in the AQP-4 knockout-induced intracerebral hemorrhage mouse model [144]. 

The nucleotide-binding oligomerization domain-like receptor pyrin domain-containing 3 (NLRP3) inflammasome has a significant role in AD-related neuroinflammation. The interaction of Aβ with astrocytes and macroglia can activate NLRP3 inflammasome, causing the release of chemokines and inflammatory mediators and activating the caspase-1 cascade [145]. The activation of NLRP3 inflammasome results in the BBB breakdown by the generated cytokines, whereas reducing the inflammasome activation reduces the inflammatory response and improves the BBB function [146]. NF-κB is another inflammatory transcription factor that is increased in AD. The activation of NF-κB by Aβ plaques, NFT, or oxidative stress causes the release of proinflammatory cytokines and ROS and promotes neuroinflammation. Like neuroinflammation, oxidative stress plays a significant role in the BBB disruption [141]. Oxidative stress is a condition produced by an imbalance between the generation and accumulation of ROS in cells and tissues and the ability of a biological system to detoxify these reactive products [147]. Neuroinflammation and oxidative stress result in neuronal cell death, altered neurotransmitter production and activity, and decreased synaptic functioning, all of which can lead to cerebral injury and dysfunction [117,148]. Oxidative stress disrupts endothelial cells by producing inflammatory responses, activating MMPs, or disrupting redox-sensitive transcription factors such as the protein Redox-factor-1 (Ref-1/Ape1) [149]. Peroxide-induced oxidative stress in an in vitro BBB model using human iPSC-derived endothelial cells caused the BBB disruption. This BBB disruption was monitored by an increased TEER and mediated by the upregulation of inflammatory mediators, modulating cell turnover and increasing immune cell adhesion [150].

In AD, Aβ deposits cause pericyte degeneration [151,152]. Aβ-pericytes signaling causes vascular constriction and decreases CBF through ROS generation in humans and rodents [153]. This degeneration is affected by the ApoE isoform where ApoEε4 accelerates the BBB breakdown; ApoEε4 also reduces the calcineurin–nuclear factor of activated T cells (NFAT) signaling in pericytes and causes the BBB disruption [154]. ApoEε4 iPSC-derived-mural cells expressed higher levels of cytoplasmic and nuclear NFATc1 protein and exhibited a higher gene expression of calcineurin catalytic subunits (PPP3CA and PPP3CC). Increased levels of NFATc1 induce the CAA pathology by interacting with ApoEε4 promoter in ApoEε4-expressing pericytes. Inhibiting calcineurin in iPSC-derived mural cells reduced ApoE expression in ApoE4-expressing cells and NFATc1 in ApoE3-expressing cells [154]. Another signaling pathway that is affected by AD pathology is the platelet-derived growth factor-BB (PDGF-BB): PDGF receptor-β (PDGFRβ) signaling pathway. In AD, the reduction in PDGF-BB: PDGFRβ signaling in brain pericytes disrupts the BBB by reducing the proliferation of pericytes. This signaling pathway protects from apoptosis through extracellular signal-regulated kinase (ERK) signaling and protects pericytes from inflammation through Akt signaling [155]. 

In AD, the dysfunction of the Wnt/β-catenin signaling pathway has been associated with the BBB breakdown. Under normal conditions, Wnt/β-catenin signaling is essential for the CNS angiogenesis [156]. Reduction in this signaling pathway reduces the expression of TJs and modulates the expression of transporters such as P-gp and GLUT1, leading to an increased BBB permeability [156]. Suppressing Wnt/β-catenin signaling in the APP_swe_/PS1_dE9_ (APP/PS1) microvessels increased glycogen synthase kinase-3 beta (GSK-3β), whereas its activation, using LRP6-optogenetic tool, restored the BBB TJs, and prevented Aβ-induced endothelial cells’ dysfunction [157]. The same effect has been shown in human iPSCs where the activation of the Wnt/β-catenin pathway using CHIR 99021 (CHIR), an inhibitor of GSK-3β and an agonist of Wnt/β-catenin, induced GLUT1 and claudin-5 expressions, and downregulated levels of plasma lemma vesicle-associated protein (PLVAP) [158]. Wnt/β-catenin signaling also regulates paracellular and transcellular permeabilities [159]. Adult mice with β-catenin endothelial-conditional knockout exhibited a reduced TJ protein expression, a downregulated MFSD2a receptor, and an increased caveolae-mediated transcytosis [159]. 

The genetic underpinnings of the BBB dysfunction in AD provide a fascinating, yet relatively understudied, angle to the ongoing research in the field. A significant proportion of genes implicated in the AD risk have been linked to the brain’s vascular and perivascular systems, further suggesting the integral role of the BBB’s integrity in the AD pathogenesis. The human brain vasculature expresses 30 out of the top 45 genes linked to the AD risk based on genome-wide association studies (GWAS) [160]. Remarkably, several genes identified in GWAS, including PICALM, known for its involvement in endocytosis facilitating the internalization of cell receptors, have variants that can increase the Aβ accumulation in the brain, exacerbating the AD pathology [161], as shown by triggering clathrin-mediated endocytosis through its interaction with LRP1 [162]. Recent investigations have posited that PICALM may protect neurons against the deleterious effects of Aβ toxicity [161]. This protective mechanism appears to operate through two distinct mechanisms: firstly, PICALM is implicated in the reversal of Aβ-induced disruptions in clathrin-mediated endocytosis [163], and secondly, it plays a role in directing the APP transport toward the terminal degradation pathway via autophagosomes [164]. Similarly, bridging integrator 1 (BIN1) and CD2-associated protein (CD2AP), both associated with vesicle trafficking and synaptic function, are believed to modulate Aβ transcytosis across the BBB, thereby influencing the AD progression [162,165]. Another gene, Ras and Rab interactor 3 (*RIN3*), also engaged in vesicle trafficking, has been connected with BBB dysfunction and its variants are linked to AD and vascular dementia [166]. 

Mutations in the OCLN gene, responsible for encoding occludin [167], and the junctional adhesion molecule-C (JAM-C) gene, responsible for encoding the junctional molecule JAM-C, are directly implicated in the compromise of the BBB’s integrity [168,169]. Furthermore, NOTCH3 gene, vital for vascular integrity through smooth muscle cell differentiation, presents mutations that affect blood vessel walls and the BBB’s integrity, further linking it to AD and vascular dementia [170]. Mice with the CADASIL-associated Notch3-R169C mutation exhibit an accumulation of the NOTCH ectodomain within pericytes. This accumulation is concomitant with the pericyte degeneration and a subsequent compromise of the BBB’s integrity [171]. Moreover, inactivating mutations in the MFSD2a gene encode the BBB transporter responsible for the uptake of essential ω-3 fatty acids, resulting in a lethal microcephaly syndrome [172]. Mice deficient in Mfsd2a demonstrate a compromised cerebral uptake of ω-3 fatty acids [173]. Concurrently, there is an observed deregulation of caveolae-mediated transcellular transport across the BBB, leading to its breakdown [173,174].

TREM2, which encodes the triggering receptor expressed on myeloid cells 2 protein, was highlighted as an AD susceptibility gene in two GWAS [175,176]. Notably, these studies identified the R47H missense mutation, which presents a heightened AD risk comparable to possessing one ApoEε4 allele [175]. Predominantly expressed in brain microglia, TREM2 is pivotal in mediating the neuroinflammatory reactions associated with AD [177]. In-depth functional analyses of TREM2 have elucidated its involvement in the modulation of Aβ plaque accumulation in brain parenchyma, the advancement of tau-related pathology, and BBB dysfunction [176,178,179,180]. TREM2 may affect the integrity of the BBB by affecting inflammation, the microglial oxidative response, and insulin resistance. Moreover, many studies have found that soluble TREM2 (sTREM2) may disrupt the BBB’s integrity in AD by interacting with pro-inflammatory proteins such as TNF receptor 1 and TNF receptor 2, and their effectors like intercellular adhesion molecule 1 and vascular cell adhesion molecule 1 [181]. The increment of these molecules affects the vascular endothelial function and the integrity of the BBB [182].

Another genetic risk factor for AD is formin-related protein 2 (FMNL2), an astroglia-expressed protein instrumental in glia–vasculature interactions and Aβ regulation [183]. Research indicated an elevated FMNL2 expression in both AD and cerebrovascular pathology [184]. Vascular risk factors amplify FMNL2 expression in AD patients, suggesting its pivotal role in exacerbating the AD pathology and vascular anomalies [185]. UNC5B is one of the members of the UNC5 family of netrin-1 receptors, which is believed to play a role in maintaining the BBB’s integrity [186]. In adult mice, the endothelial-specific removal of Unc5B results in the BBB’s permeability due to the capillary transformation into a barrier-deficient state characterized by a diminished claudin-5 and an augmented PLVAP expression. This loss of Unc5B also reduces BBB Wnt/β-catenin signaling [186]. Furthermore, data derived from a model of multiple sclerosis highlighted the downregulation of FOXO1, which was linked to changes in the BBB [187]. This finding becomes more intriguing as FOXO1 upregulation was shown to reduce the Aβ production and the tau phosphorylation in vitro [188].

## 6. The BBB as a Therapeutic Target

The BBB dysfunction has been implicated in the pathogenesis of AD and many other neurodegenerative diseases [30,189]. While there are new medications targeting Aβ, such as the recent FDA-approved monoclonal antibody Lecanemab, a curative therapy for AD remains elusive. Furthermore, these medications are associated with severe adverse reactions, including edema and cerebral microhemorrhages [190]. Such concerns underscore the potential need for combined therapies that simultaneously address the BBB function and other key aspects of AD. Accordingly, there has been a growing interest in developing therapeutic strategies that target the BBB to enhance drug delivery, improve the clearance of toxic molecules, and restore the barrier function [191]. Strategies to modulate the BBB function in AD can be broadly categorized into three main areas: enhancing the Aβ clearance across the BBB, improving the BBB’s integrity and function, and addressing neuroinflammation [30,189]. Indeed, the intricate regulation of Aβ transporters, both influx and efflux, plays a crucial role in the Aβ accumulation. The dysregulation of these transporters has been recognized as a potential therapeutic target of AD [191].

### 6.1. Enhancing Aβ Clearance across the BBB

#### 6.1.1. RAGEs 

RAGE upregulation in the vascular endothelium, neuronal cells, and microglia in the brains of AD patients highlights the crucial role of RAGEs in the pathophysiology of neurovascular dysregulation and neurodegeneration in this disorder. AD-induced endothelium-RAGE increases the Aβ influx into the brain from the blood. Accordingly, RAGE inhibitors present a promising therapeutic option for halting the disease progression in AD patients [192]. 

RAGE inhibitors have been shown to slow the Aβ pathology and decrease the rate of cognitive decline in animal models [193]. Recent research has highlighted that a specific tertiary amide, discovered through library screening, acts as a competitive antagonist against the interaction of Aβ and RAGE at the BBB [194]. This amide enhances functional CBF responses following brain stimulation and cognitive processes and diminishes brain Aβ concentrations in an AD mouse model, specifically the APPsw−/+ mice [194]. Nevertheless, a Phase III clinical trial with a RAGE inhibitor (NCT02080364) was terminated due to its low efficacy [184]. Moreover, a randomized, double-blind, placebo-controlled study was conducted to assess PF-04494700, an oral RAGE inhibitor, in patients diagnosed with mild-to-moderate AD. However, this pilot study revealed that PF-04494700 had no consistent or significant impact on the plasma Aβ levels, inflammation-related markers, or other cognitive and functional outcomes [195].

#### 6.1.2. LRP1 

The dysregulation of LRP1 in AD patients’ brain vasculature and parenchyma has implications for the clearance and accumulation of Aβ peptides, critical players in the disease’s pathogenesis [196]. LRP1 has been identified as a potential therapeutic target for AD [197]. Targeting LRP1 to enhance the Aβ clearance represents a promising strategy for mitigating the neurovascular and cognitive dysfunction observed in AD [198]. Accordingly, statins, for instance, have been tested and shown to upregulate LRP1 at the BBB and reduce brain Aβ levels [199]. Despite the lack of clear-cut efficacy in initial clinical trials, a reanalysis suggested its potential benefits in AD patients, mainly those homozygous for the ApoEε4 allele [200]. Although the role of LRP1 modulation remains not fully understood, these findings highlight the potential of targeting Aβ efflux mechanisms in AD.

Moreover, natural compounds have been identified to increase the level of LRP1 in the brain or cerebrovasculature. For example, resveratrol increases overall LRP1 levels in the mouse brain [201]. Besides its role in the Aβ clearance across the BBB, LRP1 induction induces neurogenesis and mitochondrial biogenesis by enhancing AMP-activated protein kinase (AMPK) [201].

In vitro studies demonstrated that Crocus sativus extract increases the tightness of a cell-based BBB model [202]. Furthermore, in the 5XFAD mouse AD model, treatment with Crocus sativus extract showed a significant reduction in the total Aβ levels and decreased brain Aβ deposits. The decline in Aβ levels could be explained, at least in part, by the enhanced Aβ clearance across the BBB via the upregulation of LRP1 [202]. Moreover, in vitro and in vivo studies showed the potential effect of oleocanthal, a phenolic compound in extra-virgin olive oil (EVOO), to enhance the Aβ clearance from the brain via the upregulation of LRP1 [198,203]. Expression studies of LRP1 in brain endothelial cells and isolated mouse brain microvessels following treatment with rifampicin demonstrated rifampicin as an LRP1 inducer, which resulted in an increased Aβ clearance from the brain, which in part explained its protective effect against AD [204,205].

#### 6.1.3. P-gp 

As the expression and activity of P-gp are decreased in AD, it leads to the accumulation of Aβ peptides in the brain, contributing to the formation of Aβ plaques and the progression of neurodegeneration [206]. Therefore, targeting P-gp represents another promising therapeutic alternative for AD treatment, as enhancing its expression or activity could increase the clearance of Aβ from the brain across the BBB, reducing its accumulation and ameliorating AD symptoms. Accordingly, several strategies have been explored to target P-gp. For example, tariquidar, elacridar, and zosuquidar are specific P-gp modulators. However, their clinical utility is limited due to their adverse and off-target effects [207,208,209]. A study conducted by Abuznait et al. (2013) showed that oleocanthal can increase P-gp expression and functionality in brain endothelial cells [210]. Moreover, other natural compounds, like curcumin, resveratrol, Crocus sativus extract, and quercetin, have been shown to upregulate the P-gp expression and enhance its activity [202,211,212]. These compounds might represent a safer and more accessible approach to target P-gp, although further studies are needed to confirm their efficacy and safety in AD patients. A study by Qosa and his colleagues demonstrated that the Aβ clearance is enhanced from the brain across the BBB of wild-type mice treated with rifampicin or caffeine by the upregulation of LRP1 and P-gp [205]. Experimental studies of AD mouse models have demonstrated that, when treated with pregnane X receptor (PXR) agonists, which play a pivotal role in enhancing the expression of P-gp, exhibited a pronounced efflux of exogenous Aβ, which is central to the pathology of AD [213].

### 6.2. Improving BBB Integrity and Function

#### 6.2.1. Tight and Adherence Junction Proteins 

In AD, the expression levels, phosphorylation status, or localization of junction molecules may be altered, disrupting junction integrity [214]. Addressing these changes by limiting or reversing these events can help inhibit the paracellular permeability of the BBB and restore its integrity, ultimately alleviating disease symptoms [215]. Consequently, junction molecules such as adherent junctions and TJs have been proposed as therapeutic targets due to their crucial roles in regulating the paracellular permeability [40]. 

VE-cadherin, the primary component of adherents’ junctions, is critical in maintaining vascular integrity. The loss of VE-cadherin expression at these junctions contributes to the dysfunctional vascular integrity and the development of AD [216]. Conversely, restoring paracellular expression or localizing VE-cadherin within junction areas promotes repair and leads to disease regression [217]. Limited methods exist for increasing the expression of adherent junction genes in vivo. One such process involves miR-27a, a microRNA that directly regulates VE-cadherin expression. CD5-2, a target site blocker, specifically blocks miR-27a/VE-cadherin interaction and increases VE-cadherin expression [218]. This treatment significantly reduced mouse models’ BBB permeability and cerebral cavernous malformation lesion burden [218]. Moreover, an oleocanthal-enriched diet significantly increased the VE-cadherin expression by 2.3-fold in 5xFAD mouse brains [219,220].

Most studies focus on the primary TJs in the BBB (claudin-1, -3, -5, and -12) and ZO. The restoration of TJs represents a promising therapeutic target for enhancing the BBB’s integrity and improving disease treatment. While increasing claudin-5 expression reduces the BBB’s paracellular permeability and normalizes barrier function, maintaining appropriate levels is critical [221]. In a murine AD model, breaking down TJs allows the paracellular clearance of neurotoxic Aβ peptides across the BBB, improving cognitive function. However, the controlled and targeted modulation of TJs of the BBB requires a more profound understanding before a therapeutic consideration [222].

Recently, findings from studies by our group demonstrated that, in the 5xFAD mouse model, oleocanthal administration resulted in a substantial upregulation in the expression of key BBB components: claudin-5, occludin, and ZO1. These increases were approximated at 1.9-, 1.7-, and 2.0-fold, respectively [219]. Additionally, in a subsequent study, EVOO was associated with significant enhancements in claudin-5 and occludin expressions, registering increases of 44% and 56%, respectively, in mouse models of AD [220]. In a parallel strand of research, granisetron exhibited the potential to enhance the BBB’s integrity. Experiments with bEnd3 cells revealed that granisetron augments the expression of TJ proteins and decreases intracellular calcium levels in vitro. These results were corroborated in TgSwDI mice, where granisetron enhanced the BBB’s integrity. This improved integrity was concomitantly linked with the upregulated expression of TJ proteins [223]. Furthermore, investigations into compounds like etodolac and α-tocopherol, when applied in combination at 10 μM concentrations, showcased an evident impact on the expression of claudin-5 and ZO-1, with the combination therapy yielding the most pronounced effect [224]. Moreover, a series of in-depth analyses using immunocytochemistry on bEnd3 monolayers indicated that drugs such as oxaprozin, etodolac, beclomethasone, and candesartan substantially upregulated the claudin-5 expression, highlighting the potential of these drugs in enhancing the BBB’s integrity [224].

Etodolac, α-tocopherol, and their combination significantly induced the expression of claudin-5 and ZO-1, with the combination demonstrating the highest effect on bEnd3 cells and brain microvessels of 5XFAD mice [224]. The expression and localization of claudin-5 in bEnd3 monolayer using immunocytochemistry and Western blot analysis demonstrated that oxaprozin, beclomethasone, and candesartan significantly induced the claudin-5 expression [203]. Finally, besides its effect on LRP1 and P-gp, Crocus sativus extract remarkably increased the expression of claudin-5 in the brain microvessels of 5XFAD mice, signifying its therapeutic potential in rectifying the BBB function [202].

#### 6.2.2. GLUT1 

AD’s pathogenesis has been associated with a decreased expression of GLUT1, the primary glucose transporter at the BBB. As a result, targeting GLUT1 has been proposed as a potential therapeutic approach for AD. The rationale for targeting GLUT1 in AD is to enhance glucose uptake and restore glucose metabolism in the brain, thereby facilitating cognitive deficits and neuronal dysfunction. GLP1 receptor agonists, including Liraglutide, have been shown to prevent the decline in glucose transport through the BBB in AD patients [225]. These effects are thought to occur by restoring GLUT1 levels at the BBB [225]. A recent study also suggested a reduced incidence of dementia in patients with type 2 diabetes treated with GLP1 receptor agonists [226]. This data led to a Phase IIb clinical trial (NCT01843075) that demonstrated Liraglutide’s potential to improve cognitive function and MRI volume in patients with mild-to-moderate AD [227], leading to optimism about GLP1 analogs as a potential disease-modifying therapy for AD. Furthermore, a phase III randomized controlled trial evaluating Semaglutide in early AD is underway (NCT04777396).

#### 6.2.3. MFSD2a

Targeting MFSD2a is crucial for multiple reasons. Primarily, MFSD2a mediates the transport of essential nutrients vital for brain development and function. MFSD2a is integral for maintaining the BBB’s integrity, making it a potential therapeutic target [228]. It also restricts the paracellular transport of water and ions, which helps preserve the selective permeability of the BBB, essential for its proper neuronal functioning [229]. Studies have reported that ApoE genotypes may influence the MFSD2a function. ApoEε4 carriers have been shown to have a reduced MFSD2a expression, which could lead to the BBB dysfunction and an increased susceptibility to AD [230]. In contrast, the ε2 allele may protect the BBB by promoting the MFSD2a function [231]. Given the influence of ApoE genotypes on MFSD2a function and AD risk, targeting MFSD2a could represent a potential therapeutic approach for individuals with specific ApoE genotypes. Enhancing MFSD2a expression or function may help restore the BBB’s integrity, particularly in ApoEε4 carriers at a higher risk of AD. This strategy could involve the development of small molecules or gene therapies that modulate the MFSD2a expression or activity [232]. Additionally, identifying compounds that can selectively enhance the MFSD2a function in the context of the ApoEε4 allele might offer a personalized treatment option for individuals at a higher genetic risk of AD [30].

#### 6.2.4. AQP-4

AQP-4 is a bidirectional water channel predominantly expressed in astrocytes, facilitating water transport across cell membranes. AQP-4 is also present in perivascular astrocytic endfeet, which are essential components of the BBB. Studies have revealed that AQP-4 dysregulation may contribute to age-related brain changes and play a role in AD pathogenesis. Studies have found altered AQP-4 expression and localization in AD mouse models and post-mortem human AD brains. For example, the expression of the Aqp-4 gene was increased in the cerebral and cerebellar cortices of older mice (17 months old) compared to their younger adult counterparts [233]. In line with these findings, Zeppenfeld et al. (2017) reported that changes in AQP-4 immunostaining were associated with aging in post-mortem human cortical tissues [234].

Some pharmacological agents have shown the potential to modulate AQP-4 expression and function. Phytocompounds with antioxidant properties, such as pinocembrin, curcumin, and epigallocatechin gallate, have been shown to downregulate the AQP-4 expression in various models [235,236,237]. Drugs like atorvastatin, TGN-020, and goreisan can potentially reduce brain edema through AQP-4 regulation [238,239]. New AQP-4 partial antagonists, AER-270 and AER-271, have demonstrated beneficial results in brain edema models [240]. The AQP-4 modulation may slow brain aging and prevent neurological deficits, but a further understanding of AQP-4 biology is needed to maximize the benefit and limit the toxicity of a therapy [241].

#### 6.2.5. PCSK9

Proprotein convertase subtilisin/kexin type 9 (PCSK9) is an enzyme that has garnered considerable interest in neurodegeneration [242]. PCSK9 binds to LDLRs, including LRP1, on the cell surface, instigating their degradation within lysosomes and consequently diminishing the cellular uptake of LDL cholesterol. Intriguingly, this mechanism has implications for AD, as LRP1 is instrumental in the clearance of Aβ. Recent investigations have shed light on the deleterious effects of PCSK9 on the LRP1-mediated Aβ clearance at the BBB [243]. Experimental studies employing an established BBB model have demonstrated that PCSK9 compromises the LRP1 function, leading to an attenuated brain-to-blood Aβ clearance as evidenced in vitro. Moreover, administering monoclonal anti-PCSK9 antibodies to 5xFAD mice has ameliorated the cerebral Aβ accumulation. Notably, these therapeutic benefits were absent in mice with brain endothelial-specific deletion of LRP1, underscoring the critical role of LRP1 in this process. The therapeutic antibodies targeting peripheral PCSK9 not only reduced the Aβ pathology in memory-critical regions such as the prefrontal cortex and hippocampus but also mitigated cognitive deficits associated with hippocampal function [243].

Collectively, these findings suggest that peripheral PCSK9 inhibition, leveraged by FDA-approved monoclonal antibodies, offers a promising and readily implementable strategy for AD treatment. By enhancing the Aβ clearance from the brain, PCSK9 inhibitors could potentially alter the course of AD, providing a novel avenue for intervention.

### 6.3. Targeting Neuroinflammation

The integrity of the BBB is intrinsically linked to the functional state of the associated pericytes, astrocytes, and microglia [244,245,246]. An increasing number of studies suggest that BBB impairment is consistently associated with neuroinflammation-driven neurodegeneration, which has been noted in AD patients [245,246,247]. Activated microglia and astrocytes release pro-inflammatory cytokines, such as transforming growth factor-β (TGF-β), interleukin-1 β (IL-1β), TNF-α, and IL-6, which are upregulated with the onset of pathologic processes characterized by a compromised BBB function [90,248]. Accordingly, targeting neuroinflammation could provide an effective approach for maintaining a functional BBB with a reduced risk of AD or a halt in AD progression. For example, glucocorticosteroids with anti-inflammatory and immunosuppressive effects control unwanted inflammatory responses and improve the BBB’s integrity [249]. Therapeutic agents targeting inflammation and its downstream signaling pathways, including angiogenesis, oxidative stress, and cytoskeleton reorganization, have also been explored to restore the BBB [249].

#### 6.3.1. TGF-β

TGF-β plays a multifaceted role in neuroinflammation [250]. While TGF-β can increase the paracellular permeability of vascular endothelial layers by influencing the tyrosine phosphorylation of VE-cadherin and claudin-5, it is also implicated in the deleterious cycle where a compromised BBB amplifies TGF-β signaling in astrocytes, which in turn exacerbates cognitive deficits in aging rodents [251]. Blocking TGF-β signaling can ameliorate these age-related cognitive effects in mice [252]. IL-1β is another molecule that affects the BBB’s integrity during neuroinflammation. Animal studies reveal that countering IL-1β’s effects, either by using IL-1β receptor antagonists or through the genetic removal of the IL-1 receptor, can mitigate the BBB’s hyperpermeability induced by neuroinflammation [253].

#### 6.3.2. VCAM and ICAM-1

Inflammation increases the expression of adhesion molecules such as vascular cell adhesion protein 1 (VCAM-1) and intercellular adhesion molecule 1 (ICAM-1). The upregulation of VCAM-1, in particular, has been linked with age-related cognitive deficits, making it a potential therapeutic target for neurodegeneration associated with aging [254]. One mechanism that facilitates leukocyte binding is the interaction between leukocyte-expressed integrins and VCAM-1 [255]. Natalizumab, which targets α4 integrin, prevents the interaction between α4 integrin and VCAM-1. This mechanism has been observed to restore the BBB’s integrity [256,257]. However, ICAM-1 targeting has produced varied outcomes. While the murine antibody against human ICAM-1 (enlimomab) showcased protection in animal models, its effectiveness in humans remained inconclusive, possibly due to unfavorable immune responses against the mouse-derived components of the antibody [258,259].

#### 6.3.3. NLRP3 Inflammasome

Moreover, numerous studies have shown that the activation of the NLRP3 inflammasome and the subsequent release of IL-1β are linked to the BBB damage and its increased permeability. In TgSwDI mice, EVOO significantly lowered brain IL-1β levels compared to mice administered refined olive oil. This effect was linked to reduced caspase-1 and caspase-8 expressions, key enzymes in IL-1β formation. Furthermore, EVOO’s inhibition of the NLRP3 inflammasome also decreased astrogliosis and microglial activation [146]. Additionally, MRI and Evans blue permeability tests showed a notable decrease in infarction volume, edema formation, and the BBB’s permeability in NLRP3^−/−^ ischemic mice, aligning with improved neurologic scores [260].

#### 6.3.4. AMPK and cAMP

5′-Adenosine monophosphate-activated protein kinase (AMPK) signaling plays a crucial role in maintaining the BBB’s integrity, especially under neuroinflammatory conditions [261,262]. When the AMPK activation decreases, such as during exposure to neuroinflammatory mediators like lipopolysaccharide (LPS), the BBB becomes compromised, as observed by the reduced expression of tight junction proteins like claudin-5. However, drugs like metformin, AICAR, and melatonin, which activate AMPK, have shown promise in alleviating the BBB impairments in mice models [262]. Additionally, imatinib, a small tyrosine kinase inhibitor, enhances the BBB’s integrity, reduces neuroinflammation, and targets the PDGFR-α signaling, indicating its potential efficacy in conditions like multiple sclerosis [263].

Intracellular cyclic adenosine monophosphate (cAMP) is recognized for promoting barrier-enhancing properties in endothelial cells. Interventions that elevate cAMP, such as treatment with cell-permeable cAMP analogs or phosphodiesterase inhibitors, have been observed to enhance the barrier function [264]. One notable mechanism involves cAMP’s action on BBB tight junctions, specifically upregulating claudin-5 expression independent of protein kinase A (PKA) and reinforcing the barrier by phosphorylating claudin-5 via the cAMP/PKA pathway [265]. 

#### 6.3.5. CypA-MMP-9 

The activation of the proinflammatory CypA-MMP9 pathway in pericytes has been shown to contribute to the breakdown of the BBB [231]. NF-κB transcriptionally activates MMP9 in cerebral vessels, causing the BBB dysfunction [72]. Previously, it has been shown that ApoEε4 stimulates the CypA-MMP9 pathway, which could lead to the BBB breakdown and ultimately cause neuronal and synaptic dysfunction. Studies have reported that the blockade of the CypA-MMP9 pathway in ApoEε4 knock-in mice rectifies the BBB’s integrity and restores neuronal and synaptic functions [231]. Accordingly, targeting the CypA-MMP9 cascade could represent another promising strategy [266]. In addition, the genetic inhibition of the CypA-MMP-9 pathway at the BBB reversed neurodegenerative changes in Apoe^−/−^ mice [231]. SB-3CT, an MMP9 inhibitor, eliminated the MMP9 gelatinase activity and reversed the BBB’s leakiness [231].

Furthermore, the administration of granisetron in the AD mice model TgSwDI significantly affected various aspects of neuroinflammation and neuronal function. Specifically, granisetron treatment led to a 30% decrease in the microglial marker Iba-1 and a 47% reduction in MMP-9 levels. This effect by granisetron was associated with decreased neuroinflammation, increased expression of neuro-synaptic markers, reduced apoptosis, and elevated acetylcholine levels in the brain [223].

## 7. Conclusions

AD presents a formidable challenge in drug development, as reflected by a staggering 99% failure rate in clinical trials. The reasons for this attrition are deeply rooted in the intricate pathophysiology of AD and the complexity of its treatment. The BBB, a critical regulator of cerebral homeostasis, has emerged as a nexus of AD pathology. Its dysfunction not only precedes cognitive symptoms but also offers a potential target for therapeutic intervention. Understanding the nuanced interplay between the BBB breakdown and the AD progression is key for developing strategies that either bolster the BBB defenses or exploit its permeability to deliver treatments effectively.

The diversity of genetic and pathological factors in AD, such as the variability in Aβ peptides and the early onset of neuronal damage, complicates the development of effective treatments. Drugs targeting Aβ have been particularly challenging due to the structural heterogeneity of Aβ and the difficulty in delivering therapeutic agents across the BBB. Monoclonal antibodies, like aducanumab, have shown the potential of reducing Aβ deposits, but not without risks highlighting the need for a balance between efficacy and safety. Moreover, misdiagnosis has been another hurdle, with symptomatic assessments often misidentifying patients, thereby complicating clinical trial results. This has led to a shift in focus towards biological markers, i.e., Aβ deposition, tau pathology, and neurodegeneration, validated by imaging and biofluid tests for more accurate diagnosis. However, these methods are expensive, can expose patients to high radiation levels, or require invasive procedures, spurring the search for safer, more affordable, and more precise diagnostics such as blood tests. Accordingly, the field is pivoting towards the prevention and the deceleration of neurodegeneration, particularly in those predisposed to AD. Combination therapies that target multiple pathways, along with innovative non-pharmacological interventions, such as non-invasive surgical techniques, are gaining traction. Furthermore, emphasizing a healthy lifestyle can delay the onset of AD, potentially eradicating it for many individuals.

Insights gleaned from past trials underscore the urgent need to preserve and restore the BBB function as a cornerstone of innovative strategies aimed at mitigating the progression and severity of AD. This review not only sheds light on the complex interplay between AD and the BBB but also highlights the transformative impact of employing the BBB as a therapeutic target. By integrating these perspectives, we are poised to make significant strides in the management of AD, offering the prospect of slowing disease progression to enhance the quality of life for individuals with AD, and lay the foundation for future research and treatment strategies that harness the BBB function as a barrier and its use as a potential target for therapy. 

## Data Availability

Not applicable.
